# Editorial: Women in psychiatry 2022: Addictive disorders

**DOI:** 10.3389/fpsyt.2023.1157677

**Published:** 2023-04-11

**Authors:** Wendy J. Lynch, E. Blair Towers, Emilie F. Rissman

**Affiliations:** ^1^Psychiatry and Neurobehavioral Sciences, University of Virginia, Charlottesville, VA, United States; ^2^Medical Scientist Training Program, University of Virginia, Charlottesville, VA, United States; ^3^Center for Human Health and the Environment, North Carolina State University, Raleigh, NC, United States

**Keywords:** substance use disorder, women, opioids, editorial, gender bias

Numbers of women entering the field of academic psychiatry [52.4%; (1)] are on the increase. Women now represent over half of medical students [50.7%; (2)], neuroscience and psychology graduate students [up to 80%; (3)], psychiatry residents [57%; (4)], and post-docs [53%; (5)] in the United States. The proportion of NIH early-career awards to women has also gone up over the last 30 years; particularly over the past 6 years (6). These award mechanisms include pre-doctoral fellowships (F30/31), post-doctoral fellowships (F32), mentored research career awards (K01/7/8/22/23/25/99, KL1/2), and appointment on Kirschstein-NRSA training grants (T32/34/35/36/90, TL1/4, TU2). The proportion of women authoring peer-reviewed papers was approaching fifty percent as of 2018 (7).

However, the pipeline is stalled when it comes to keeping women in science and medicine. Women remain underrepresented among basic science and medical school faculty, particularly at advanced levels, such as among tenured faculty [42.5%; (8)], full professors [32.5%; (8)], and department chairs [18%; (9)], along with members of editorial boards of leading scientific journals in medicine and psychiatry [36%; (10)], which exert considerable power over what is published. Additionally, women post-docs and faculty continue to be paid less than men, and receive lower funding amounts (or dollars) for research grants compared to their male colleagues (5, 6, 11). Women are also under-represented as senior authors on publications and take significantly longer than men to transition from contributing to corresponding author on publications (7). This is an important indicator of independence, and given that publishing and academic success are inherently linked, may translate to the underrepresentation of women in the higher echelons of medical and basic science departments and universities.

In addition to these issues, COVID-19 appears to have disproportionately affected the productivity and scientific output of women vs. men in academia and even reversed some of the positive trends mentioned above. For example, the gender gap in the corresponding authorship position appears to have widened by an additional 10% during the pandemic (12, 13). One likely explanation for this is that during COVID-19 women assumed increased care-giving and home-schooling responsibilities as childcare and schools were shut down during the pandemic. This conclusion is supported by the literature on the division of childcare between women and men [(14, 15); also see (16)]. Thus, long-term investments in and promotion of gender equality are still needed, and if left unaddressed, could reverse or stall the gains made by women over the past several decades. Clark and Horton (17) suggested that the internal structures of both funding agencies and journals such as *The Lancet* need to be re-organized with the specific needs of women in mind in order to address gender biases in academic psychiatry. While gender biases are observed across many different fields within psychiatry, it is notable that within the addiction field, the long-standing trend for greater funding success rates of men vs. women on renewal applications through the National Institute of Drug Abuse has narrowed over the past decade, and, as of 2019, is equivalent between the genders (18).

The aim of this edition of “*Women in psychiatry 2022: addictive disorders*” of *Frontiers in Psychiatry* is to promote valuable contributions of women in the field. Four articles are included, each featuring women scientists as first or senior author. Two of these articles are focused on the impact of COVID-19 on substance use and substance use disorder. The other two examine other aspects of opioid and other substance use in human populations.

In the first article, Brown et al. report on changes in overdose deaths as the result of synthetic opioids, such as fentanyl, after the onset of COVID-19 lockdowns which began in March 2020. They found that while overdose deaths markedly increased across the United States (as compared to pre-pandemic in June 2019), by the end of the study period (November 2021), rates of overdoses plateaued in the majority of the reporting states (in 29 of the 39 states analyzed). The highest plateau was observed in Western states and in 10 states rates of overdose were still on the rise, not yet reaching maximum plateaus (i.e., Alaska, Colorado, Hawaii, Wyoming, Washington, South Dakota, Georgia, Oklahoma, Vermont, and Maine).

In the second article, Malandain et al. explore gender differences in the impact of COVID-19 lockdowns on alcohol, tobacco, and illicit drug use, internet use, and mental health in France. They found that of 263 men and women, 20% reported an increase in alcohol use, whereas 26% reported a decrease in alcohol use. For tobacco, 7% reported an increase in use, whereas 24% reported decreased use. Only 1% reported an increase in illicit drug use (such as cannabis), whereas 28% reported a decrease. Depression, anxiety, and internet use (social media, gambling, and cybersex), all increased in the same period (reported to be up in 26, 30, and 14% of participants, respectively). Surprisingly, gender was not associated with changes in any of the variables.

The third article by Washburn et al. explores knowledge and attitudes about opioid use and addiction among individuals in the Cooperative Extension System (Extension). The Extension is a US-wide network of professionals that provide community-based health education and outreach. Their role has recently been expanded, through substantial federal investments, to respond to the opioid epidemic. They focused on Extension professionals in Tennessee and showed that 90% of the 236 respondents felt that they did not possess adequate knowledge to address the opioid epidemic in their community. Respondents were mixed on their views of punitive approaches for opioid use and addiction, and while most viewed addiction as an illness (~79%), only a minority of respondents (35%) supported laws to protect people from criminal charges for drug crimes if seeking help for themselves or others experiencing a drug overdose. The authors argue that additional efforts that increase knowledge and decrease stigma associated with opioid use and addiction are necessary in order for the Extension to effectively address the opioid epidemic.

In the fourth article, Tschampl et al. explore associations between adverse childhood experiences (i.e., abuse, neglect, and household disfunction) and risk of drug overdose in individuals (140) in Massachusetts seeking treatment for substance use disorder in a predominantly Latinx population. They observed significant associations between adverse childhood experiences and risk of overdose such that each experience was associated with a 1.3 times higher risk of overdose. Women also reported more adverse childhood experiences than men.

These articles highlight some of the silent costs of social isolation and adverse childhood experiences on drug use and addiction. The contributions offer guidance as we continue to experience limitations on social interactions during COVID-19 and other public health epidemics.

## Author contributions

WJL and EBT wrote the editorial. WJL, EBT, and EFR contributed to the review of this editorial. WJL and EFR edited the Research Topic. All authors contributed to the article and approved the submitted version.
